# Why the Proximal Splenic Artery Approach is the Ideal Approach for Laparoscopic Suprapancreatic Lymph Node Dissection in Advanced Gastric Cancer? A Large-Scale Vascular-Anatomical-Based Study

**DOI:** 10.1097/MD.0000000000000832

**Published:** 2015-05-08

**Authors:** Rui-Fu Chen, Chang-Ming Huang, Qi-Yue Chen, Chao-Hui Zheng, Ping Li, Jian-Wei Xie, Jia-Bin Wang, Jian-Xian Lin, Jun Lu, Long-Long Cao, Mi Lin

**Affiliations:** From the Department of Gastric Surgery, Fujian Medical University Union Hospital, No. 29 Xinquan Road, Fuzhou 350001, Fujian Province, China.

## Abstract

Laparoscopic gastrectomy with D2 lymph node (LN) dissection has not yet been widely adopted for advanced gastric cancer because it is technically complicated. Due to the high suprapancreatic lymph nodes metastasis rate (LMR) and the various vascular anatomies, the suprapancreatic LN dissection is a crucial and demanding procedure for radical resection of gastric cancer.

To explore the anatomical basis of the proximal splenic artery (SA) approach for laparoscopic suprapancreatic LN dissection and its application in advanced gastric cancer.

Laparoscopic suprapancreatic LN dissections were performed in 1551 consecutive advanced gastric cancer patients between June 2007 and November 2013. A total of 994 consecutive patients since January 2011 were selected to compare the clinicopathological characteristics and surgical outcomes between the conventional approach group (330) and the proximal SA approach group (664). In the proximal SA approach, the No. 11p LNs are dissected first, followed by the Nos. 9, 7, and 8a LNs; dissection of the Nos. 5 and 12a LNs is performed last.

In the suprapancreatic arteries, the proximal SA had the lowest anatomic variation rate (*P* < 0.05, each) and maximum diameter (*P* < 0.05, each) compared with the common hepatic artery (CHA), left gastric artery (LGA), right gastric artery (RGA), and gastroduodenal artery (GDA). In addition, the proximal SA was located closer to the suprapancreatic border than the CHA (*P* = 0.000). The No. 11p LMR was lower than the Nos. 9, 7, 8a, 5, and 12a LMR (*P* < 0.01, each). Compared with the conventional approach, the proximal SA approach was associated with less blood loss (*P* < 0.05), significantly more retrieved total LNs and suprapancreatic LNs (*P* < 0.01, each).

The proximal SA exhibits the most constant and maximum diameter, is located closer to the suprapancreatic border, and exhibits the lowest LMR; therefore, the proximal SA approach is the ideal approach for laparoscopic suprapancreatic LN dissection in advanced gastric cancer.

## INTRODUCTION

Laparoscopic surgery is widely used in early gastric cancer because of several advantages such as reduced blood loss, reduced postoperative pain and rapid recovery^1–3^ and has been increasingly used in advanced gastric cancer over the last few years.^4^ However, laparoscopic suprapancreatic lymph node (LN) dissection along the distribution of the suprapancreatic arteries in radical gastrectomy is technically difficult because of the limited two-dimensional visualization, no sensation of touch, the complicated anatomy of the suprapancreatic vessels and the high lymph node metastasis rate (LMR) in the suprapancreatic area.^5–8^ To overcome these difficulties, several approaches have been proposed for laparoscopic suprapancreatic LN dissection in early gastric cancer,^9–11^ but because of the high suprapancreatic LMR and difficult exposure in advanced gastric cancer, to date, there has not been a vascular-anatomical-based study on the ideal approach for laparoscopic suprapancreatic LN dissection in advanced gastric cancer. Regarding advanced gastric cancer, the proximal splenic artery (SA) approach was adopted in our center for laparoscopic suprapancreatic LN dissection, in which the No. 11p LNs are dissected first, followed by the Nos. 9, 7, and 8a LNs; dissection of the Nos. 5 and 12a LNs is performed last. The study was aimed at retrospectively analyzing the features of the suprapancreatic arteries and LN metastases and the surgical outcomes in advanced gastric cancer to explore the anatomical basis for the proximal SA approach for laparoscopic suprapancreatic LN dissection and its application in advanced gastric cancer.

## METHODS

### Patients

A total of 1551 consecutive patients diagnosed with advanced gastric cancer underwent laparoscopic surgery between June 2007 and November 2013 at the Department of Gastric Surgery, Fujian Medical University Union Hospital, Fuzhou, China. The clinicopathological characteristics, surgical outcomes, and information on perigastric vascular anatomy were prospectively collected and retrospectively analyzed according to the surgical video and gastric cancer databases. When our center started the practice of laparoscopic radical resection for gastric cancer since 2007, we adopted conventional approach for suprapancreatic LN dissection. With more operation experiences and better understanding of the suprapancreatic vascular anatomies, we gradually proposed the proximal SA approach, which is routinely used in all of the patients who underwent suprapancreatic LN dissection since January 2012. To exclude the influence of the learning curve on the surgical outcomes, 994 consecutive patients with advanced gastric cancer who underwent laparoscopic suprapancreatic LN dissection after January 2011 were selected and divided into the conventional approach group (n = 330) and the proximal SA approach group (n = 664); the clinicopathological characteristics and surgical outcomes were compared between the groups. The LN grouping was according to the Japanese Classification of Gastric Carcinoma: 3rd English edition,^12^ and the tumor staging was determined according to the 7th edition of the Union for International Cancer Control (UICC) tumor, node, and metastasis (TNM) staging system.^13^ All patients and their families were informed of the study, and written informed consent was obtained for the publication of this report and any accompanying images. This study was approved by the Institutional Review Board, Fujian Medical University Union Hospital.

### Surgical Techniques

#### Conventional Approach

The conventional approach for laparoscopic suprapancreatic LN dissection was similar to that reported in the literature.^14^ After the duodenum was transected, the bifurcations of the common hepatic artery (CHA), gastroduodenal artery (GDA), and the proper hepatic artery (PHA) were exposed. And then, the Nos. 5 and 12a LNs were dissected first. Second, the CHA and the proximal SA were skeletonized in sequence to dissect the Nos. 5 and 12a LNs. The dissection of the Nos. 7 and 9 LNs was performed last, until then, the suprapancreatic LN dissection was finished.

#### The Proximal SA Approach

**FIGURE 1:**
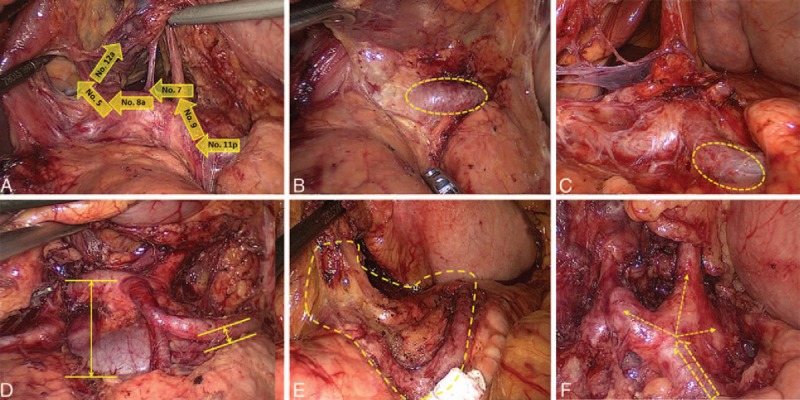
The advantages of the proximal SA approach for suprapancreatic lymph node dissection. (A) The sequence of suprapancreatic lymph node dissection; (B and C) the proximal splenic artery is revealed on the left side of the gastropancreatic fold; (D) the proximal splenic artery is close to the suprapancreatic border; (E) there are few vascular branches in the proximal splenic artery area; (F) it is convenient to locate the celiac axis, left gastric artery and common hepatic artery along the proximal splenic artery.

In the proximal SA approach, the proximal SA is first exposed to dissect the No. 11p LNs, and the suprapancreatic LNs are dissected from the left to the right side (Figure 1). The advantages are as follows: the proximal SA is associated with the most constant and maximum diameter and is located closer to the suprapancreatic border so that it is easy to expose; after exposure of the proximal SA, locating the celiac axis (CA), the left gastric artery (LGA) and the CHA is convenient for the surgeon; and in the suprapancreatic LNs, the No. 11p LMR is the lowest so that it is easy to dissect the LNs in this area and simultaneously create favorable conditions for the complete dissection of the suprapancreatic LNs.

The patient is usually placed in the supine position with the legs separated, and the surgical table is declined approximately 10°–20° in reverse Trendelenburg. The surgeon and the assistant stand on the patient's left and right sides, respectively, and the camera operator stands between the patient's legs. The 5-port method is generally used.^15^ The proximal SA approach for laparoscopic suprapancreatic LN dissection has the following features: no duodenal transection; retraction of the liver by the gastrohepatic ligament; and exposure and division of the vessels from the posterior aspect of the stomach. The No. 11p LNs are dissected first; then, the assistant places the free omentum on the left upper side of the abdomen between the inferior border of the liver and the anterior gastric wall and turns the greater curvature of the gastric body over to the cephalic side using a right-handed grasper. The surgeon gently presses down on the body of the pancreas with a gauze pad on the uppermost point of its surface to create tension on the gastropancreatic fold; in this way, the suprapancreatic area is extended to facilitate the LN dissection. Using an ultrasonic scalpel, the pancreatic capsule is meticulously peeled along the pancreatic surface up to the superior border, and the gastropancreatic fold is opened to allow entry into the retropancreatic space. Next, the assistant's right hand pulls the free pancreatic capsule up from the left side of the gastropancreatic fold, whereas the surgeon continues to peel it to separate and expose the proximal SA. Subsequently, the surgeon uses the ultrasonic scalpel to meticulously dissect the lymphatic tissue along the SA until the origin of the posterior gastric artery is reached to complete the dissection of the No. 11p LNs. Next, the Nos. 9, 7, 8a LNs, 5, and 12a LNs are dissected in sequence from the root of the SA.^16^

### Suprapancreatic Arteries

The arteries associated with suprapancreatic LN dissection are primarily the proximal SA, CHA, LGA, right gastric artery (RGA), and GDA. The abnormal proximal SA, CHA, LGA, RGA, and GDA are regarded as variant arteries including origin, course, and branch variations. All patients preoperatively underwent 64-slice spiral CT examinations (Discovery CT750HD). In addition, 2 radiologists and 2 surgeons evaluated the anatomical variations of the perigastric vessels according to the preoperative CT and operative videos, and the three-dimensional reconstructions were made using the original scanning images.

### Measurement of the Distance and Diameter

The distances from the arterial center to the suprapancreatic border was calculated by multiplying the number of slices and film pitches (0.6 mm) using MDCT consecutive thin-slice images. The suprapancreatic border was regarded as the baseline, and when the artery was above it, the distance was designated with a plus sign, but when the artery was below the border, the distance was designated with a minus sign.

The arterial diameters were measured in the arterial midpoints based on the 3D-CT images using the work station.

### Statistical Analyses

All statistical analyses were performed using the SPSS 19.0 statistical software. The measurement data are presented as the mean ± SD. Unpaired Student's *t* test and chi-square tests were used to compare the continuous variables and categorical variables. *P* < 0.05 was assumed to indicate a statistically significant difference.

## RESULTS

### Suprapancreatic Arterial Anatomies

**FIGURE 2:**
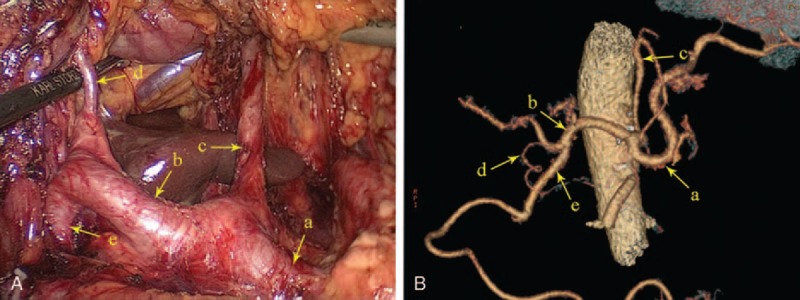
The suprapancreatic arteries, intraoperative image (A) and three-dimensional reconstruction image (B). (a) The proximal splenic artery; (b) common hepatic artery; (c) left gastric artery; (d) right gastric artery; (e) gastroduodenal artery.

The anatomical information from the suprapancreatic arteries is shown in Table 1 and Figure 2.

1. The proximal SA: the total variation rate of the proximal SA was 7.0% (108/1551), with 99 cases (6.4%) of variant origins. The course of the proximal SA is relatively constant because retropancreatic and intrapancreatic courses occurred in only 9 cases.
2. The CHA: the total variation rate of the CHA was 10.5% (163/1551). There were 81 cases with variant origins but normal courses, 15 cases with normal origins but variant courses, 6 cases with variant origins and courses, 50 cases with variant branches (branched off left hepatic artery [LHA], right hepatic artery [RHA], or RGA directly), and the other 11 cases with CHA absence and lack of replaced CHA.
3. The LGA: the total variation rate of the LGA was 16.6% (258/1551). There were 84 cases with variant origins but normal branches, 159 cases with normal origins but variant branches, 15 cases with variant origins and branches, and no cases with variant courses.
4. The RGA: the total variation rate of the RGA was 19.1% (296/1551). There were 292 cases with variant origins, 4 cases with RGA absence, and no cases with variant courses or branches.
5. The GDA: the total variation rate of the GDA was 9.7% (150/1551). There were 36 cases with variant origins but normal branches, 7 cases with variant origins and branches, 3 cases with GDA absence, and 104 cases with normal origins and variant branches.

**TABLE 1:**
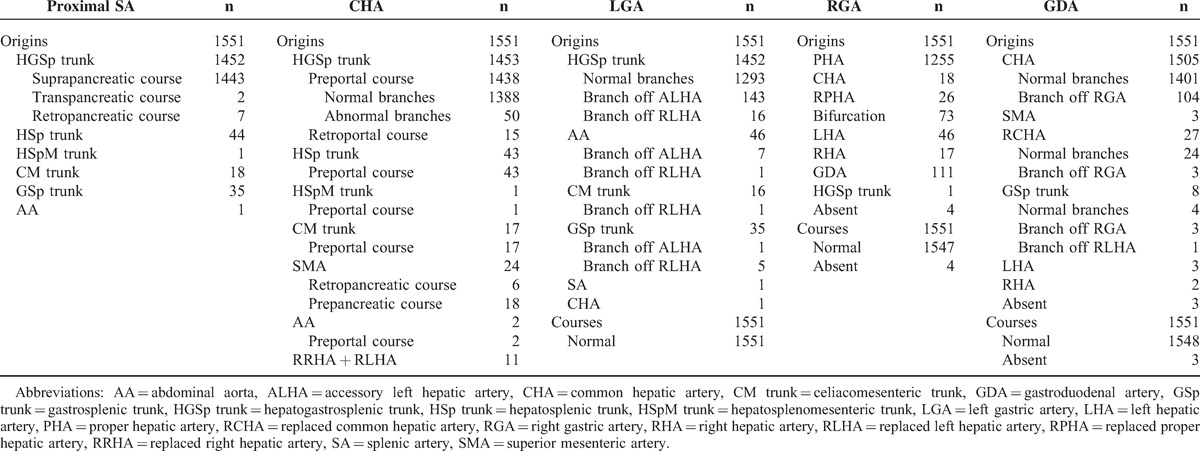
Anatomical Information of the Suprapancreatic Arteries

Compared with the CHA, LGA, RGA, and GDA, the anatomic variation rate of the proximal SA was the lowest (7.0% vs. 10.5%, 16.6%, 19.1%, and 9.7%, *P* < 0.05, each).

### Comparisons of the Diameter of Suprapancreatic Arteries

The diameter of the proximal SA was larger than the CHA, LGA, RGA, and GDA (5.5 ± 1.0 *vs.* 4.5 ± 0.9, 3.3 ± 0.6, 3.1 ± 0.5, 2.1 ± 0.4 mm, *P* < 0.05, each).

### Comparisons of the Distances from Arteries to the Suprapancreatic Border

The proximal SA and CHA were both above the suprapancreatic border, but the distance of the proximal SA to the suprapancreatic border was significantly shorter than the CHA (+0.39 ± 1.50 vs. +9.91 ± 5.81 mm, *P* = 0.000).

### Metastasis of Suprapancreatic LNs

**FIGURE 3:**
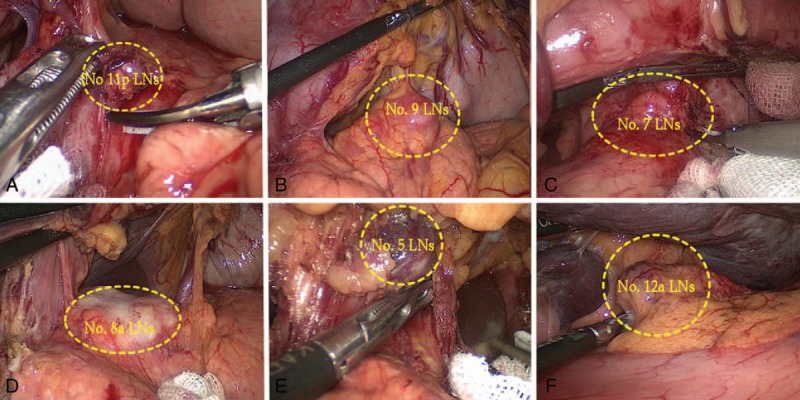
The swollen lymph nodes (LNs) in the suprapancreatic area. (A) No. 11p LNs; (B) No. 9 LNs; (C) No. 7 LNs; (D) No. 8a LNs; (E) No. 5 LNs; (F) No. 12a LNs.

As displayed in Table 2, the total LMR was 78.7% (1220/1551), and the suprapancreatic LMR (Nos. 11p, 9, 7, 8a, 5, and 12a) was 51.8% (804/1551); the No. 11p LMR was lower than the Nos. 9, 7, 8a, 5, and 12a LMR (7.0% *vs.* 23.2%, 35.4%, 18.7%, 16.1%, and 14.3%, *P* < 0.05, each). Figure 3 shows the swollen LNs in the suprapancreatic area.

**TABLE 2:**
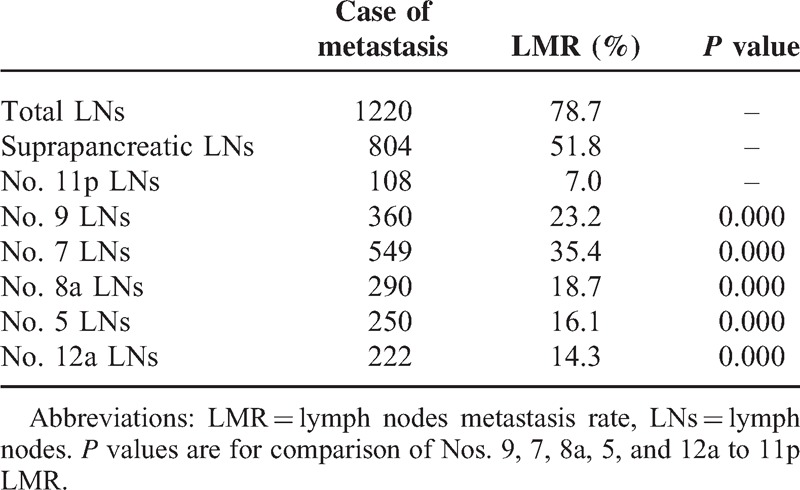
Metastases of the Suprapancreatic LNs

### The Clinicopathological Characteristics and Surgical Outcomes

Table 3 indicates that the patients’ clinicopathological characteristics had no statistically significant difference between the conventional approach group and the proximal SA approach group (*P* > 0.05, each), except for tumor location. Laparoscopic gastrectomy with D2 lymphadenectomies were successfully performed in all patients of the two groups, and a higher proportion of patients in the proximal SA approach group underwent total gastrectomies and Roux-en-Y reconstructions because the tumors were located in the upper stomach (*P* < 0.05, each). The operation time and intraoperative blood transfusion rate were similar between the groups (*P* > 0.05, each). Compared with the conventional approach, however, the proximal SA approach was associated with less blood loss (69.3 ± 95.0 vs. 86.2 ± 147.1 ml, *P* < 0.05), significantly more retrieved total LNs (38.7 ± 14.1 vs. 31.2 ± 11.7, *P* = 0.000) and suprapancreatic LNs (13.0 ± 6.2 vs. 11.7 ± 6.2, *P* = 0.002). The patients’ postoperative morbidity and mortality (*P* > 0.05, each) as well as the postoperative pancreatic leakage rate (*P* > 0.05, each) did not show any statistical significance.

**TABLE 3:**
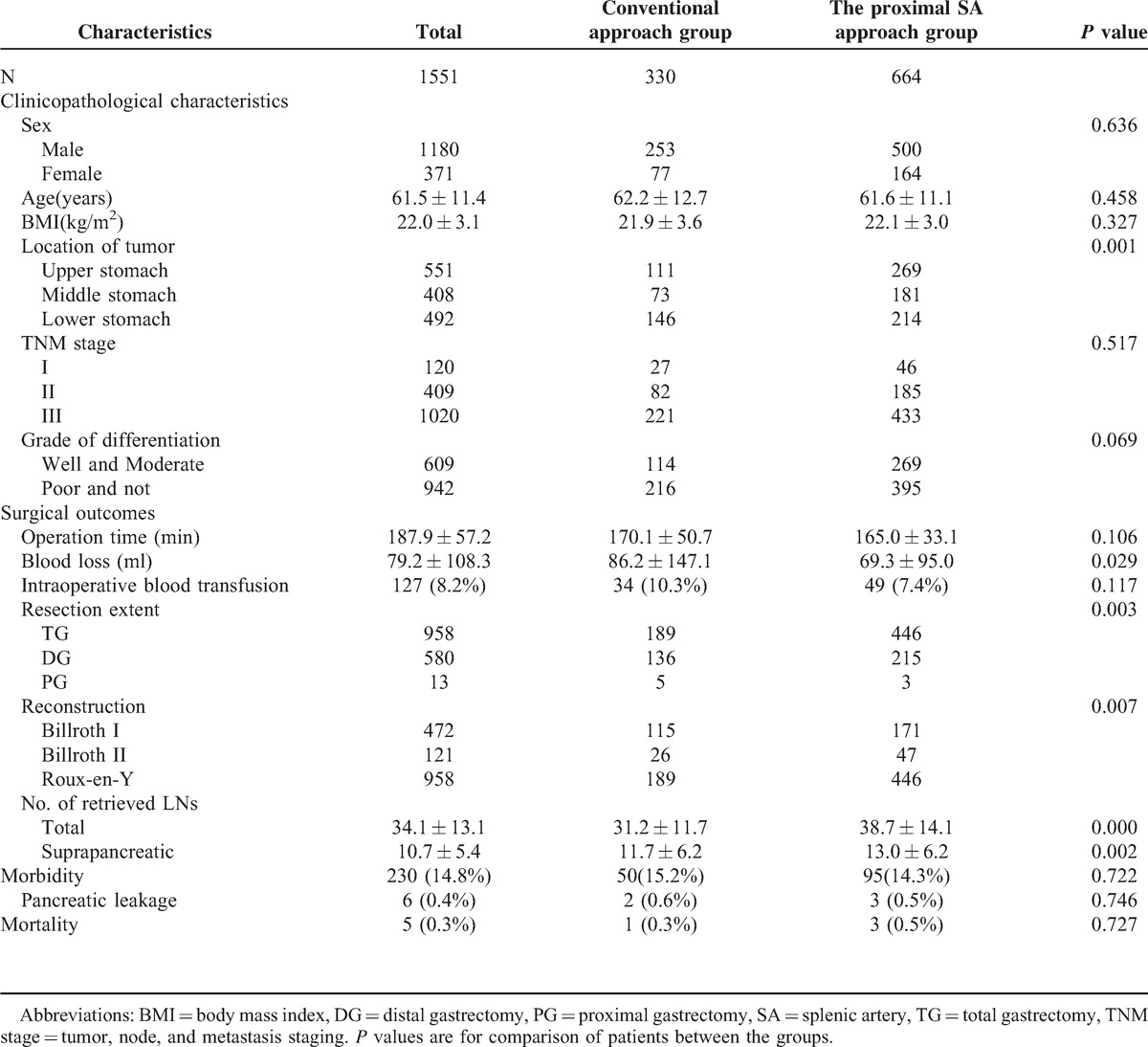
Clinicopathological Characteristics and Surgical Outcomes

## DISCUSSION

Because of the improvement in the instruments and surgical techniques, laparoscopic surgery has been becoming increasingly popular in advanced gastric cancer,^17,18^ but laparoscopic suprapancreatic LN dissection is technically demanding, and bleeding is easily caused;^5^ therefore, these cases sometimes have to be converted to open surgery. Hence, exploring a safe and feasible approach for successfully performing laparoscopic suprapancreatic LN dissection has become the hot topic at present. Techniques for taping the CHA and SA were adopted to dissect the LNs along the CHA and SA in open surgery. If similar techniques are used in laparoscopic surgery,^14,19,20^ the technique is difficult and fewer LNs are retrieved compared to open surgery.^21,22^ The LMR in the suprapancreatic area is relatively low in early gastric cancer;^23–25^ hence, laparoscopic surgery is associated with less blood loss and clearer visualization than open surgery. Regarding early gastric cancer, the feasibility of the left-side approach for laparoscopic suprapancreatic LN dissection was reported by Fukunaga et al^9^; this approach was associated with several advantages such as clear visualization, less bleeding, low morbidity, suitability for obesity, and ease of the technique.^26^ The medial approach for laparoscopic suprapancreatic LN dissection was first reported by Kanaya et al,^10^ and the conveniences and feasibilities in patients without vascular variation has been reported in the literature.^27^ However, the LMR in the suprapancreatic area is quite high in advanced gastric cancer, and swollen LNs often occur during LN dissection. In addition, the surgeon may cut into the LNs incorrectly resulting in vestigial LNs. LNs are fragile, and clamping or lifting the LNs directly can easily cause bleeding and tumor cell dissemination. In addition, lymph leakage easily occurs from ruptured LNs leading to the retention of lymphatic fluid, which negatively affects the surgical field. In addition, the swollen LNs always wrap round the vessels, which increases the difficulty of vascularizing the vessels. Therefore, laparoscopic suprapancreatic LN dissection is more difficult for advanced gastric cancer than for early gastric cancer. For advanced gastric cancer, there is still no consensus on the most suitable approach for laparoscopic suprapancreatic LN dissection.

Through retrospective analyses of the anatomic features in suprapancreatic arteries, we found that not only was the proximal SA closer to the suprapancreatic border and the incidence of the proximal SA variations was the lowest but also the diameter of the proximal SA was the largest, so that the SA is easy to expose. If the proximal SA is treated as the approach, the surgeon can gain entry into the anatomic space anterior to the artery quickly and accurately, and the CA, LGA, and CHA will be revealed step-by-step when dissecting the adipose tissue along the space anterior to the artery toward the right side. After the left side of the suprapancreatic border with the lower vascular variation rate is adequately isolated, the right side of the suprapancreatic border with the higher vascular variation rate will be revealed sufficiently by the assistant's traction by means of the un-transected duodenum, which is useful for successfully accomplishing the suprapancreatic LN dissection and reducing blood loss. However, in the cases in this study, the CHA was away from the suprapancreatic border, and the variation rate was up to 10.5%; if the CHA is regarded as the approach, the space posterior to the CHA may be entered, which increases the risk of the portal vein (PV) injury. In addition, if the CHA is absent or located posterior to the PV, not only is it difficult to locate the suprapancreatic arteries such as the LGA, the proximal SA and the CA along the PV, but it is also easy to enter the incorrect anatomic space and cause vascular damage. In addition, as revealed in the results of our study, the LMR around the proximal SA is the lowest, so that it is likely to reduce LN bleeding and lymphatic fluid leakage when the proximal SA is isolated first, to maintain clear vision, which makes it easier to dissect the LNs in this area and create favorable conditions for the complete dissection of the suprapancreatic LNs. With the proximal SA approach, the No. 11p LNs are dissected first, followed by the Nos. 9, 7, and 8a LNs; dissection of the Nos. 5 and 12a LNs is accomplished last, so that the suprapancreatic LNs were dissected *en bloc*, which is effective for not only reducing blood loss but also increasing the number of retrieved suprapancreatic LNs. In this study, the surgical outcomes between the conventional approach group and the proximal SA approach group were compared, and the results indicate that the operation time was similar (*P* > 0.05); however, the proximal SA approach was associated with less blood loss (*P* < 0.05) and significantly more retrieved total LNs and suprapancreatic LNs (*P* < 0.01, each). Furthermore, the postoperative short-term outcomes were satisfactory.

In summary, the proximal SA is associated with the most constant and maximum diameter, is located closer to the suprapancreatic border and exhibits the lowest LMR, so that the proximal SA approach is the ideal approach for laparoscopic suprapancreatic LN dissection in advanced gastric cancer. Nevertheless, our study is limited by its single-center nonrandomized retrospective research, and more reliable results should be obtained in multicenter randomized prospective studies.

## References

[1] Sakuramoto S Yamashita K Kikuchi S Laparoscopy versus open distal gastrectomy by expert surgeons for early gastric cancer in Japanese patients: short-term clinical outcomes of a randomized clinical trial. *Surg Endosc* 2013; 27:1695–1705. 23247737 10.1007/s00464-012-2658-9

[2] Zeng YK Yang ZL Peng JS Laparoscopy-assisted versus open distal gastrectomy for early gastric cancer: evidence from randomized and nonrandomized clinical trials. *Ann Surg* 2012; 256:39–52. 22664559 10.1097/SLA.0b013e3182583e2e

[3] Adachi Y Shiraishi N Shiromizu A Laparoscopy-assisted Billroth I gastrectomy compared with conventional open gastrectomy. *Arch Surg* 2000; 135:806–810. 10896374 10.1001/archsurg.135.7.806

[4] Goh PM Khan AZ So JB Early experience with laparoscopic radical gastrectomy for advanced gastric cancer. *Surg Laparosc Endosc Percutan Tech* 2001; 11:83–87. 11330389

[5] Uyama I Kanaya S Ishida Y Novel integrated robotic approach for suprapancreatic D2 nodal dissection for treating gastric cancer: technique and initial experience. *World J Surg* 2012; 36:331–337. 22131088 10.1007/s00268-011-1352-8

[6] Natsume T Shuto K Yanagawa N The classification of anatomic variations in the perigastric vessels by dual-phase CT to reduce intraoperative bleeding during laparoscopic gastrectomy. *Surg Endosc* 2011; 25:1420–1424. 20976496 10.1007/s00464-010-1407-1

[7] Adachi Y Shiraishi N Suematsu T Most important lymph node information in gastric cancer: multivariate prognostic study. *Ann Surg Oncol* 2000; 7:503–507. 10947018 10.1007/s10434-000-0503-1

[8] Methasate A Trakarnsanga A Akaraviputh T Lymph node metastasis in gastric cancer: result of D2 dissection. *J Med Assoc Thai* 2010; 93:310–317. 20420105

[9] Fukunaga T Hiki N Tokunaga M Left-sided approach for suprapancreatic lymph node dissection in laparoscopy-assisted distal gastrectomy without duodenal transection. *Gastric Cancer* 2009; 12:106–112. 19562465 10.1007/s10120-009-0508-9

[10] Kanaya S Haruta S Kawamura Y Video: laparoscopy distinctive technique for suprapancreatic lymph node dissection: medial approach for laparoscopic gastric cancer surgery. *Surg Endosc* 2011; 25:3928–3929. 21660629 10.1007/s00464-011-1792-0

[11] Satoh S Okabe H Kondo K Video. A novel laparoscopic approach for safe and simplified suprapancreatic lymph node dissection of gastric cancer. *Surg Endosc* 2009; 23:436–437. 18528615 10.1007/s00464-008-9978-9

[12] Sano T Aiko T New Japanese classifications and treatment guidelines for gastric cancer: revision concepts and major revised points. *Gastric Cancer* 2011; 14:101–112. 21573921 10.1007/s10120-011-0040-6

[13] Sobin LH Gospodarowicz MK Wittekind C TNM classification of malignant tumours. New York: John Wiley & Sons; 2011.

[14] Uyama I Sugioka A Matsui H Laparoscopic D2 lymph node dissection for advanced gastric cancer located in the middle or lower third portion of the stomach. *Gastric Cancer* 2000; 3:50–55. 11984710 10.1007/pl00011690

[15] Jia-Bin W Chang-Ming H Chao-Hui Z Laparoscopic spleen-preserving No. 10 lymph node dissection for advanced proximal gastric cancer in left approach: a new operation procedure. *World J Surg Oncol* 2012; 10:241–248. 23146045 10.1186/1477-7819-10-241 PMC3502297

[16] Huang CM Chen QY Lin JX Laparoscopic suprapancreatic lymph node dissection for advanced gastric cancer using a left-sided approach. *Ann Surg Oncol* 2015; 21:2051. 25608768 10.1245/s10434-014-4309-y

[17] Shinohara T Satoh S Kanaya S Laparoscopic versus open D2 gastrectomy for advanced gastric cancer: a retrospective cohort study. *Surg Endosc* 2013; 27:286–294. 22733201 10.1007/s00464-012-2442-x

[18] Uyama I Suda K Satoh S Laparoscopic surgery for advanced gastric cancer: current status and future perspectives. *J Gastric Cancer* 2013; 13:19–25. 23610715 10.5230/jgc.2013.13.1.19 PMC3627802

[19] Tanimura S Higashino M Fukunaga Y Laparoscopic gastrectomy with regional lymph node dissection for upper gastric cancer. *Br J Surg* 2007; 94:204–207. 17058319 10.1002/bjs.5542

[20] Uyama I Sugioka A Fujita J Completely laparoscopic extraperigastric lymph node dissection for gastric malignancies located in the middle or lower third of the stomach. *Gastric Cancer* 1999; 2:186–190. 11957094 10.1007/s101200050044

[21] Miura S Kodera Y Fujiwara M Laparoscopy-assisted distal gastrectomy with systemic lymph node dissection: a critical reappraisal from the viewpoint of lymph node retrieval. *J Am Coll Surg* 2004; 198:933–938. 15194075 10.1016/j.jamcollsurg.2004.01.021

[22] Memon MA Khan S Yunus RM Meta-analysis of laparoscopic and open distal gastrectomy for gastric carcinoma. *Surg Endosc* 2008; 22:1781–1789. 18437472 10.1007/s00464-008-9925-9

[23] Lee HH Yoo HM Song KY Risk of limited lymph node dissection in patients with clinically early gastric cancer: indications of extended lymph node dissection for early gastric cancer. *Ann Surg Oncol* 2013; 20:3534–3540. 23846783 10.1245/s10434-013-3124-1

[24] Bravo Neto GP dos Santos EG Victer FC Lymph node metastasis in early gastric cancer. *Rev Col Bras Cir* 2014; 41:11–17. 24770768 10.1590/s0100-69912014000100004

[25] Ren G Cai R Zhang WJ Prediction of risk factors for lymph node metastasis in early gastric cancer. *World J Gastroenterol* 2013; 19:3096–3107. 23716990 10.3748/wjg.v19.i20.3096 PMC3662950

[26] Kitano S What technique is suitable for laparoscopic suprapancreatic lymph node dissection? *Gastric Cancer* 2009; 12:67–68. 19562459 10.1007/s10120-009-0512-0

[27] Shinohara T Hanyu N Kawano S Clinical significance of medial approach for suprapancreatic lymph node dissection during laparoscopic gastric cancer surgery. *Surg Endosc* 2014; 28:1678–1685. 24380991 10.1007/s00464-013-3370-0

